# Comparison the accuracy and trending ability of cardiac index measured by the fourth-generation of FloTrac with the PiCCO device in septic shock patients

**DOI:** 10.3906/sag-1909-58

**Published:** 2020-06-23

**Authors:** Bodin KHWANNIMIT, Rattina JOMSURIYA

**Affiliations:** 1 Division of Critical Care Medicine, Department of Internal Medicine, Prince of Songkla University, Hat Yai, Songkhla Thailand

**Keywords:** Cardiac output, hemodynamic monitoring, pulse contour analysis, arterial waveform analysis, measurement technique

## Abstract

**Background/aim:**

FloTrac/Vigileo is a noncalibrated arterial pressure waveform analysis for cardiac index (CI) monitoring. The aim of our study was to compare the CI measured by the 4th generation of FloTrac with PiCCO in septic shock patients.

**Materials and methods:**

We simultaneously measured the CI using FloTrac (CIv) and compared it with the CI derived from transpulmonary thermodilution (CItd) as well as the pulse contour-derived CI using PiCCO (CIp).

**Results:**

Thirty-one septic shock patients were included. The CIv correlated with CItd (r = 0.62, P < 0.0001). The Bland-Altman analysis showed a bias of 0.14, and the limits of agreement were –1.62–1.91 L/min/m2 with a percentage error of 47.4%. However, the concordance rate between CIv and CItd was 93.6%. The comparison of CIv with CIp (n = 352 paired measurements) revealed a bias of -0.16, and the limits of agreement were –1.45–1.79 L/min/m2 with a percentage error of 44.8%. The overall correlation coefficient between CIv and CIp was 0.63 (P < 0.0001), and the concordance rate was 85.4%.

**Conclusion:**

The 4th generation of FloTrac has not acceptable agreement to assess CI; however, it has the ability to tracked changes of CI, when compared with the transpulmonary thermodilution method by PiCCO.

## 1. Introduction

The cardiac index (CI) is recommended as one of the most important hemodynamic variables for the assessment of cardiac function, and guidance of therapy in critically ill patients within an intensive care unit (ICU) [1]. The measurement of CI, via the pulmonary artery catheter (PAC), is presently the standard method; however, the data of increasing morbidity [2, 3] associated with its use has intensified the development of a less invasive device for advanced cardiac monitoring. For instance, transpulmonary thermodilution as implemented in the PiCCO device has been developed [4]. Previous studies showed that CI measured by this device is comparable with that by the PAC [5–7]. However, this device required initial calibration to access the hemodynamic parameters [8].

The noncalibrated arterial pressure waveform analysis (FloTrac/Vigileo, Edwards Lifescience, Irvine, CA, USA) has been proposed for continuous CI monitoring. This device is widely used for monitoring critically ill patients in operating rooms and ICUs, due to being less invasive and without needing external calibration. However, the reliability of CI measured by this device has had conflicting results. Some studies presented that CI obtained by the FloTrac is reliable as CI measured by PAC [9,10]; in contrast, other studies have revealed a limited correlation, or agreement with other reference CI devices [4,11,12], especially during hyperdynamic states [13,14] or hemodynamic instability [15,16]. So, the software of FloTrac was regularly updated for improved performance of this device. The 3rd generation software is able to recognize many hyperdynamic and vasoplegia conditions, even though the accuracy and tracking ability of this version are still controversial [17–20]. Recently, the 4th generation of FloTrac software extended the autocalibration factor to adjust for acute changes in the systemic vascular resistance (SVR). Previous literatures reported the 4th generation of FloTrac had been significantly improved in its ability to track the changes in CI induced by phenylephrine or increased vasomotor tone compared with the previous version [21,22]. In contrast, some studies presented that the 4th generation of FloTrac still lacks accuracy along with trending ability in cardiac surgical patients [23,24]. However, the validation of CI measured by the 4th generation of FloTrac has been limited evaluation for septic shock patients. 

Thus, the aim of our study was to validate the accuracy of the 4th generation of FloTrac with the PiCCO (transpulmonary thermodilution and pulse contour analysis) as a reference technique in patients with septic shock. The secondary aim was to determine the tracking ability in CI obtained by the latest version of FloTrac, induced by either increasing the dosage of norepinephrine or fluid bolus therapy. 

## 2. Material and methods 

### 2.1 Patients

This prospective study was conducted in the Medical Intensive Care Unit of Songklanagarind Hospital, Prince of Songkla University, Thailand. It was approved by the Institutional Human Research Ethics Committee (HREC) (54-042-14-1-2), and informed written consents were obtained from the next of kin of each patient. Subjects were informed as soon as their mental status allowed, and the possibility was given for them to withdraw from the study.

The inclusion criteria were as follows: 1. Having septic shock, as defined by the Sepsis–3 definition [25]; 2. Existence of a central venous catheter. The patients were excluded if they had intracardiac shunts and cardiac arrhythmias (atrial fibrillation or frequent premature beats), due to impaired reliability of the transpulmonary thermodilution method and pulse contour analysis.

### 2.2 Study protocol

For all patients, the PiCCO 5 French 20 cm arterial thermistor catheter (Pulsiocath PV2015L20, Pulsion Medical Systems, Munich, Germany) was inserted into the femoral artery. The arterial line was divided into 2 branches, 1 connected to the Philips CCO/CO module (model M10212A, Philips Medical Systems, Böblingen, Germany) with the PiCCO software integrated into the patient’s monitor, Philip IntelliVue MP70 (CMS monitor model M1097A, software version 17.62, Philips Medical Systems), and the other branch connected to a FloTrac/Vigileo device (FloTrac version 04.00, Edwards Lifesciences, Irvine, CA, USA). This enabled the 2 devices to analyze the same arterial pressure waveform simultaneously. 

The FloTrac analyzed the arterial pressure waveform 100 times/s over 20 s. The stroke volume (SV) was based on the contribution of pulse pressure relative to SV, which is the proportion of pulse pressure to the standard deviation of arterial pulse pressure (APsd). The device calculated SV as APsd × X, where X compensates for differences in vascular compliance and resistance. In the 4th generation of FloTrac, X is calculated as K4 × Kfast. Kfast is inversely proportional to arterial pressure and calculated every 20 s. K4 uses multivariate polynomial equations of waveform variables such as skewness and kurtosis and is averaged every minute [21,22]. At the beginning of each data recording session, the Vigileo monitor was initialized by entering the patient’s characteristics (sex, age, weight, and height), along with zeroing the FloTrac sensor against atmospheric pressure. 

Hemodynamic measurements included recording of heart rate (HR), arterial blood pressure, central venous pressure (CVP), transpulmonary thermodilution CI (CItd), pulse contour CI (CIp), FloTrac/Vigileo CI (CIv), systemic vascular resistance index (SVRI), and other transpulmonary hemodynamic variables. The CItd was measured by the Philips CCO/CO device by injecting 15 mL of iced saline (<8 °C) through the central venous catheter. 

The CItd was calculated using the following equation:

CItd = Vi × (Tb-Ti) × k/AUC ÷ BSA

When Vi is the injected volume, Tb is blood temperature, Ti is injected temperature, k is a constant proportional to the specific weights and specific heat of blood and injected, AUC is the area under the transpulmonary thermodilution curve and BSA is body surface area [26]. 

The injection was manually performed in triplicate, and the values of CItd were averaged. The CItd was repeated every 8 h for 48 h.

The CIp was estimated by the following equation:

CIp = cal × HR × × dt 

When cal is the patient specific calibration factor determined with thermodilution, is area under the pressure curve, C(p) is compliance, is shape of pressure curve.

We used the values of CIp and CIv that were automatically displayed on the screens of both devices. The CIv and CIp were recorded every 2 h over a period of 48 h, after study initiation. 

We evaluated the tracking trends in CIv compared with CIp induced by either increasing the dosage of norepinephrine (n = 56), or fluid bolus (n = 16) in the subgroups of septic shock patients. Hemodynamic data were collected before and after therapeutic interventions (at 5 min after increase norepinephrine or at the end of fluid bolus).

### 2.3 Statistical analysis

The normal distribution of data was tested by the Shapiro-Wilk test. The correlation was assessed by Pearson’s coefficient. The level of agreement as well as bias between the methods were evaluated by Bland-Altman analysis and corrected with repeated measurement [27]. The percentage errors were calculated as: 1.96 times of standard deviation of the bias divided by the mean CI of reference methods [19,20,28,29]; a percentage error less than 45% was considered clinically acceptable [30,31]. The trending ability of CI was assessed by 4-quadrant plot analysis, with an exclusion zone of 10% [22]. For this method, the concordance rate was defined as the proportion of the number of paired CI change, with the same direction of changes in both methods, which were presented in the upper right and lower left quadrant. A concordance rate of more than 90% was defined as an acceptable value [32]. In addition, we analyzed significant influence on bias between CIv and CItd, using a linear mixed effect model for repeated measurements. Mean arterial pressure (MAP), SVRI, and norepinephrine dose were treated as fixed effects while each patient was treated as a random effect. Comparisons between subjects receiving fluid bolus and an up dose of norepinephrine were performed by a 2–tailed Student t test or Mann-Whitney U test, as appropriate. We compared the relative changes of CIv with those of CIp during the therapeutic intervention by the Bland-Altman analysis and correlation analysis. A receiver operating characteristic (ROC) curve was used to assess the ability of CIv to detect concordant and significant CIp ≥ 15%, after both interventions. A 2–sided P value < 0.05 was considered statistically significant. Statistical analysis was performed by Stata software version 11.

## 3. Results

Thirty-one mechanically ventilated septic shock patients were enrolled in our study. Patient characteristics are summarized in Table 1. All patients received norepinephrine administration and 13 patients (41.9%) were treated with combined vasoactive agents at the time of inclusion in our study. 

**Table 1:** Patients clinical characteristic (n = 31)

Men, n (%)	17 (54.8)
Age (years)	56.3 ± 18.5
Body weight (kg)	60.6 ± 10.1
Height (cm)	161.8 ± 8.2
Body surface area (kg/m2)	1.64 ± 0.16
APACHE II	26.5 ± 8.1
SOFA	9.8 ± 3.7
ICU length of stay (days)	7.7 ± 5.4
Community acquired infection, n (%)	20 (64.5)
Site of infection, n (%)	
- Respiratory tract infection	13 (41.9)
- Digestive system	7 (22.6)
- Primary bacteremia	4 (12.9)
- Others*	7 (22.5)
Combined vasoactive, n (%)	13 (41.9)
Norepinephrine (ug/kg/min)	0.33 ± 0.18
Dopamine (ug/kg/min)	11.5 ± 10.3

APACHE, acute physiology and chronic health evaluation; ICU, intensive care unit; SOFA, sequential organ failure assessment

### 3.1 Comparison between CIv and CItd

**Figure 1:**
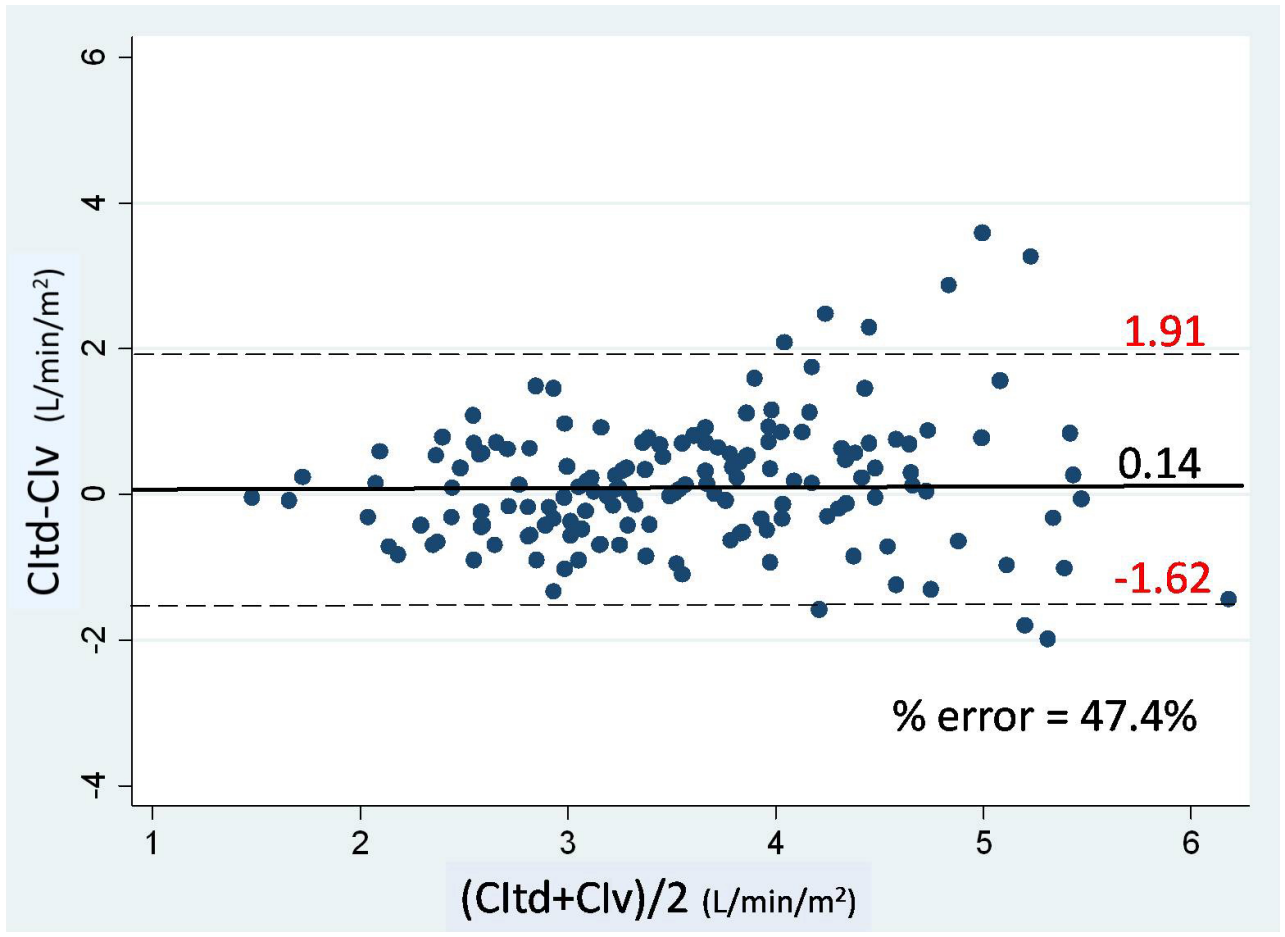
Bland-Altman plot for CIv and CItd. Straight line indicating mean bias, dash line indicating 95% limit of agreement CIv, cardiac index obtained by FloTrac; CItd, cardiac index measured by transpulmonary thermodilution of PiCCO.

**Figure 2:**
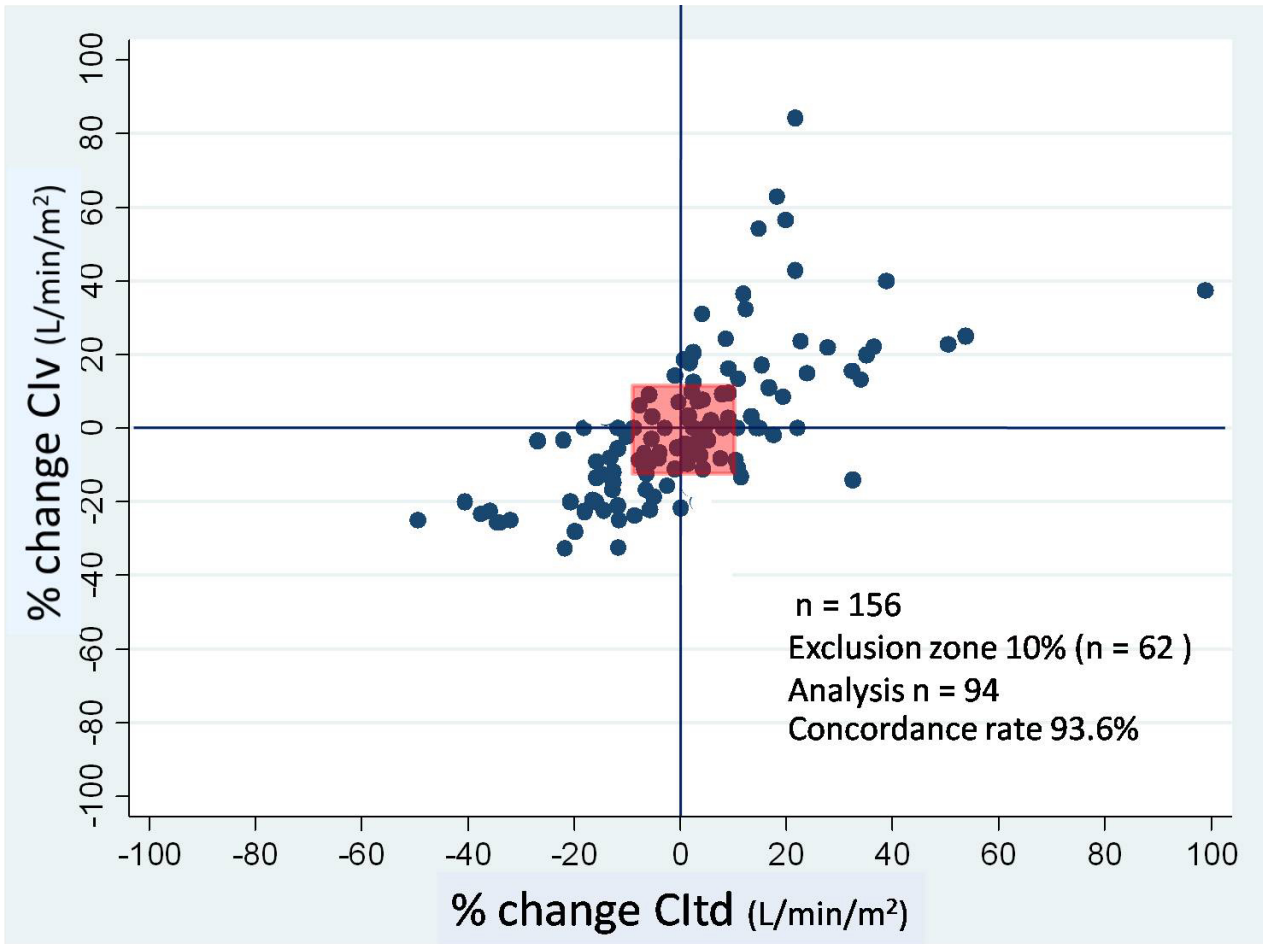
Four-quadrant plot analysis to determine the trending ability for percentage change of CIv against CItd. Central square is an exclusion zone (% change < 10%); CIv, cardiac index obtained by FloTrac; CItd, cardiac index measured by transpulmonary thermodilution of PiCCO.

There were 156 paired CI measurements obtained to compare between CIv and CItd. CIv and CItd ranged from 1.5–6.8 and 1.5–6.9 L/min/m2 respectively. The CIv was significantly correlated with CItd with r = 0.62 (P < 0.0001) (Table 2). Bland-Altman analysis showed that bias along with limits of agreement was 0.14 and –1.62–1.91 L/min/m2 with the percentage error being 47.4% (Figure 1). Figure 2 presents the 4–quadrant plot analysis between CIv and CItd, which has an acceptable concordance rate of 93.6%. The bias of CIv and CItd was correlated with SVRI (r = -0.46, P < 0.0001). The linear mixed effect model for repeated measurements, showed that the bias between CIv and CItd was influenced only by SVRI (P < 0.001).

**Table 2:** Summary of the comparison between cardiac index measured by the 4th generation of FloTrac with PiCCO.

	Correlation, r	Bias(L/min/m2)	Limits of agreement(L/min/m2)	Percentageerror (%)	Concordance (%)
CIv-CItd (n = 156)	0.62	0.14	–1.62–1.91	47.4	93.6
CIv-CIp (n = 352)	0.63	-0.16	–1.45–1.79	44.8	85.4

CIv, cardiac index obtained by FloTrac.CIp, cardiac index measured by pulse contour analysis of PiCCO.

### 3.2 Comparison of CIv with CIp

**Figure 3:**
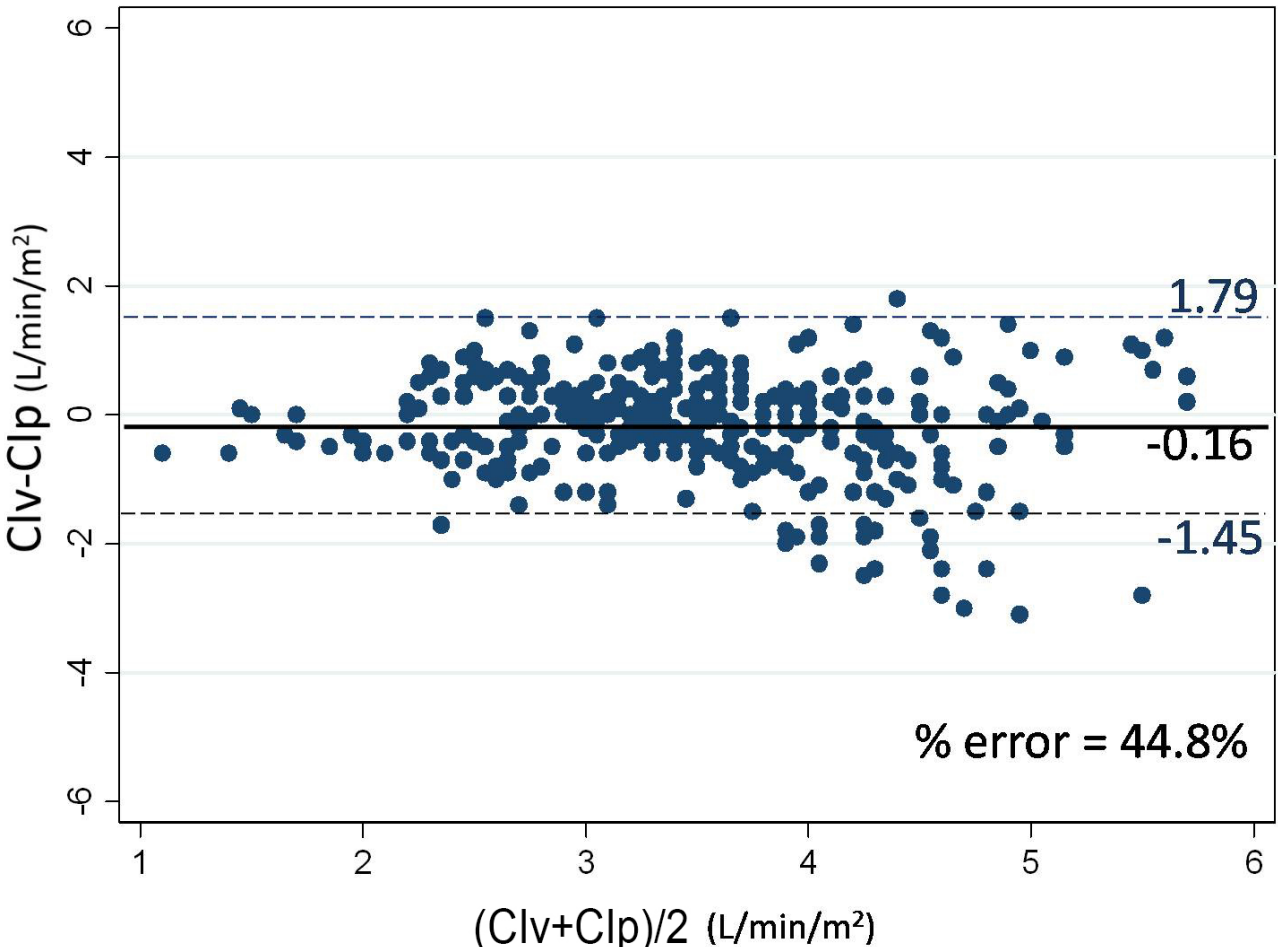
Bland-Altman plot for CIv and CIp. Straight line indicating mean bias, dash line indicating 95% limit of agreement CIv, cardiac index obtained by FloTrac; CIp, cardiac index measured by pulse contour analysis of PiCCO.

**Figure 4:**
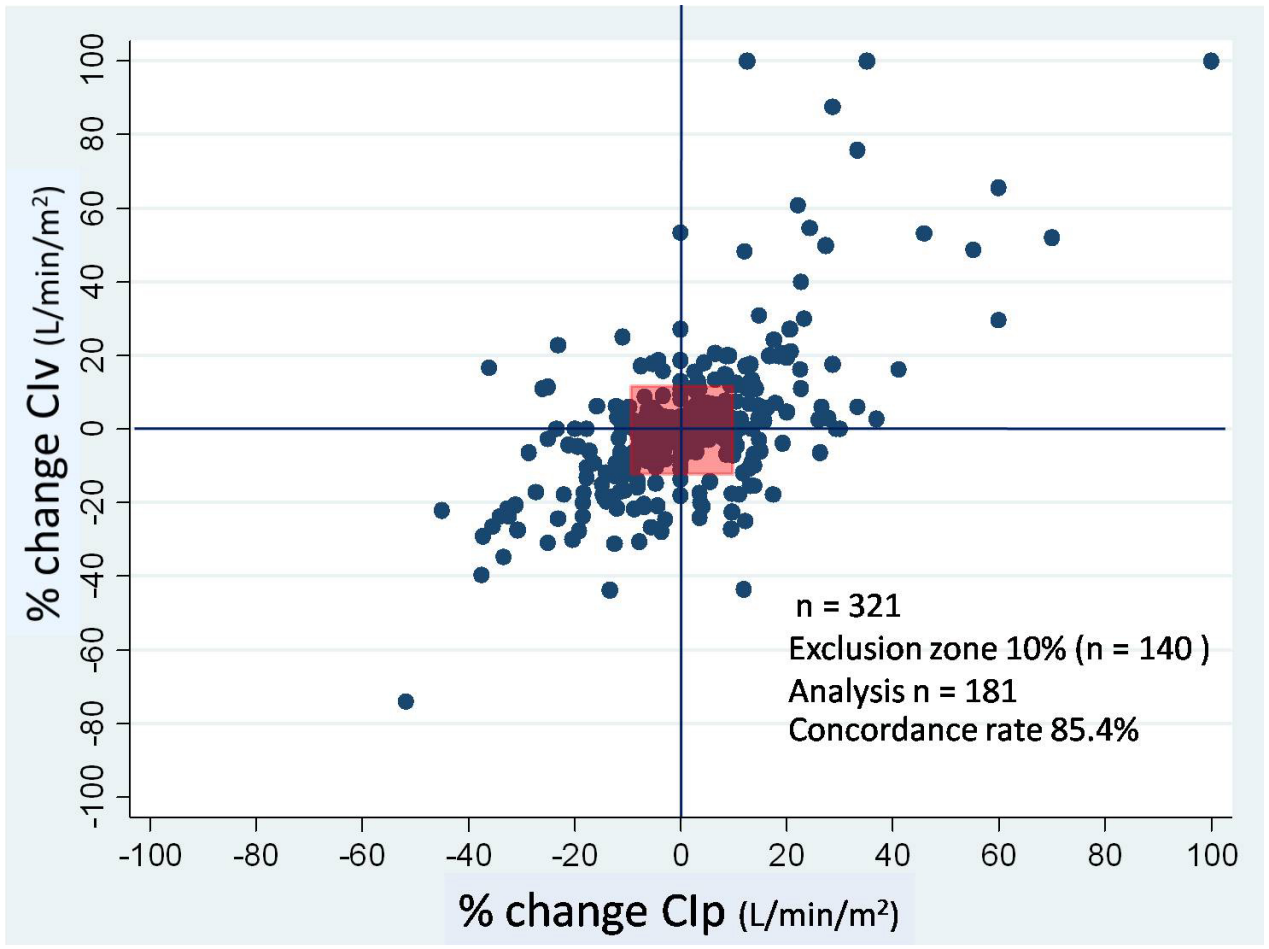
Four-quadrant plot analysis to determine the trending ability for percentage change of CIv against CIp. Central square is an exclusion zone (% change <10%) CIv, cardiac index obtained by FloTrac; CIp, cardiac index measured by pulse contour analysis of PiCCO.

A total of 352 paired CI measurements were obtained, so as to compare between the 2 methods. CIv ranged from 1–6.2, while CIp ranged from 1.4–6.9 L/min/m2. A significant correlation was observed between CIv and CIp (r = 0.63, P < 0.0001) (Table 2). The Bland-Altman corrected, for repeated measurements, showed bias of –0.16 and limits of agreement were –1.45–1.79 L/min/m2 with a percentage error of 44.8% for CIv and CIp (Figure 3). The concordance rate between CIv and CIp was 85.4% (Figure 4). 

### 3.3 Comparison of CIv and CIp in subjects with an increased dose of norepinephrine

**Figure 5:**
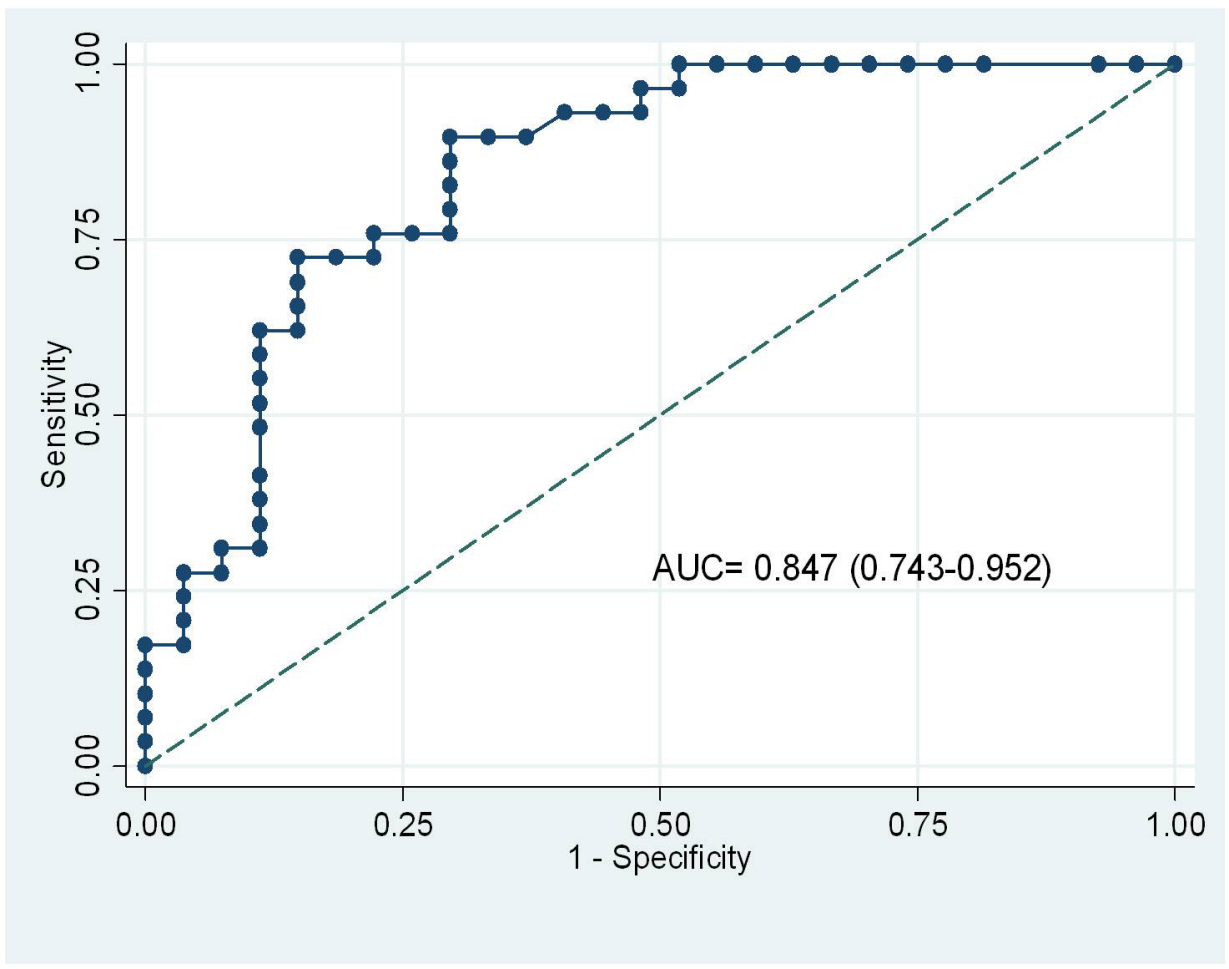
Receiving operating characteristic curves for the changes in CIv to detect an increase in CIp ≥ 15% induced by increase dose of norepinephrine. CIv, cardiac index obtained by FloTrac; CIp, cardiac index measured by pulse contour analysis of PiCCO.

There were 56 hemodynamic data (30 patients) obtained before and after an increase in norepinephrine infusion. After increasing the dosage of norepinephrine, HR, blood pressure, CVP, CIv, and CIp were significantly increased, while SVRI and SVV were significantly decreased (Table 3). The bias and limits of agreement between the absolute changes in CIv and CIp, induced by increasing the dosage of norepinephrine were –0.47 and –1.73– 0.8 L/min/m2. The coefficient of correlation coupled with concordance rate between the percent changes in CIv and in CIp was 0.8 (P < 0.0001) and 95.8% respectively. An increase in CIv ≥ 17.5% detected an increase in CIp (≥ 15%), after an increased dose of norepinephrine with a sensitivity of 89.7% and specificity of 70.4% (AUC 0.847, 95%CI 0.743–0.952) (Figure 5).

**Table 3:** Hemodynamic parameters before and after increase dose of norepinephrine (n = 56).

	Before	After	P value
HR (/min)	105.9 ± 24.9	115.4 ± 27.2	< 0.001
SBP (mmHg)	104 (98–113)	123.5 (110.25–140)	< 0.001
DBP (mmHg)	55 (52–62)	66.5 (61–70.75)	< 0.001
MAP (mmHg)	68.4 ± 8.5	86.5 ± 11.0	< 0.001
CVP (mmHg)	14 (12–16.7)	15 (13–18)	< 0.001
SVRI (dyne-s-m2/cm5)	1,530.3 ± 410.6	1,508.3 ± 453.2	<0.001
CIv (L/min/m2)	3.2 ± 0.7	3.9 ± 0.8	< 0.001
CIp (L/min/m2)	3.4 ± 0.9	3.9 ± 0.8	0.003
SVV (%)	13 (7–22.2)	9.5 (6.2–19)	0.04
NE (ug/kg/min)	0.20 ± 0.16	0.27 ± 0.17	< 0.001

CIv, cardiac index obtained by FloTrac; CIp, cardiac index measured by pulse contour analysis of PiCCO; CVP, central venous pressure; DBP, diastolic blood pressure; HR, heart rate; MAP, mean arterial pressure; NE, norepinephrine; SBP, systolic blood pressure; SVRI, systemic vascular resistance index; SVV, stroke volume variation.

### 3.4 Comparison of CIv and CIp in subjects with receiving fluid bolus

**Figure 6:**
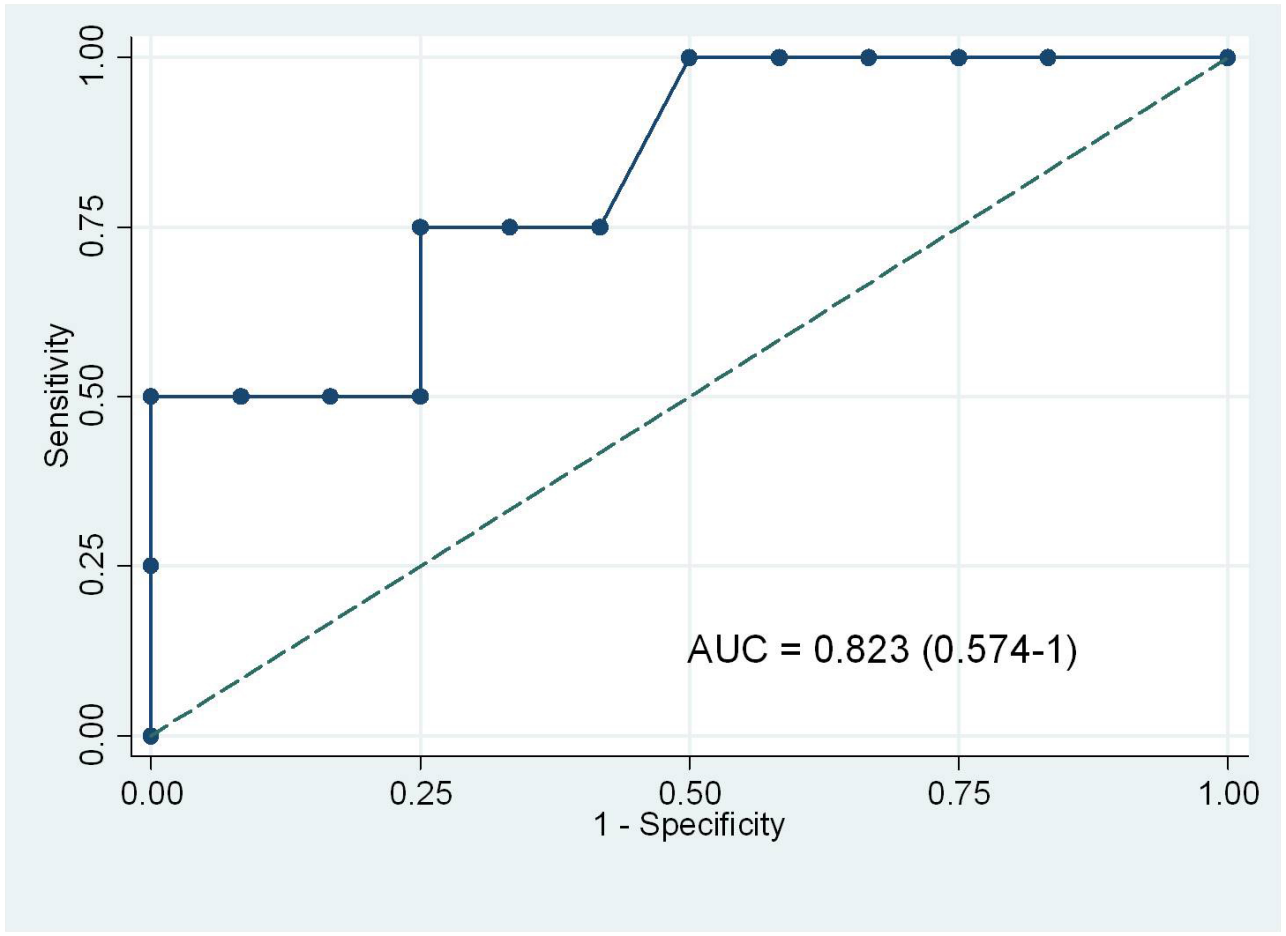
Receiving operating characteristic curves for the changes in CIv to detect an increase in CIp ≥ 15% induced by fluid loading. CIv, cardiac index obtained by FloTrac; CIp, cardiac index measured by pulse contour analysis of PiCCO.

A total of 16 hemodynamic measurements (11 patients) were performed before and after saline 500 mL bolus over 30 min. MAP, CIv, and CIp significantly increased by 10%, 14.5%, and 10.3% respectively (Table 4). The bias and limit of agreement between absolute changes in CIv and CIp induced by volume expansion were 0.05 and –0.28 to 0.38 L/min/m2. The coefficient of correlation and concordance rate between the percent changes in CIv and in CIp induced by fluid expansion were 0.49 (P = 0.05) and 83% respectively. An increase in CIv ≥ 13% detected an increase in CIp (≥ 15%) induced by fluid bolus with a sensitivity of 75% and specificity of 66.7% (AUC 0.823, 95%CI 0.574–1) (Figure 6).

**Table 4:** Hemodynamic parameters before and after volume expansion (n = 16).

	Before	After	P-value
HR (/min)	107.9 ± 24.2	104.6 ± 23.3	0.01
SBP (mmHg)	100.6 ± 22.5	110.6 ± 23.8	< 0.001
DBP (mmHg)	54 ± 8.6	57 ± 9.3	< 0.001
MAP (mmHg)	67.9 ± 13.4	75.6 ± 14.5	< 0.001
CVP (mmHg)	13.7 ± 3.1	15.2 ± 3.8	0.05
SVRI (dyne-s-m2/cm5)	1,728.1 ± 884.7	1,817.6 ± 965.9	0.29
CIv (L/min/m2)	2.7 ± 0.9	3.1 ± 0.9	0.0001
CIp (L/min/m2)	2.9 ± 1.1	3.2 ± 1.0	0.003
SVV (%)	18.5 ± 8.0	15.1 ± 8.2	0.06

CIv, cardiac index obtained by FloTrac; CIp, cardiac index measured by pulse contour analysis of PiCCO; CVP, central venous pressure; DBP, diastolic blood pressure; HR, heart rate; MAP, mean arterial pressure; NE, norepinephrine; SBP, systolic blood pressure; SVRI, systemic vascular resistance index; SVV, stroke volume variation.

## 4. Discussion

Our results found that the 4th generation of FloTrac has no clinically acceptable agreement to estimate CI, but correctly tracked changes in CI when compared with transpulmonary thermodilution method by PiCCO. In addition, the 4th generation of FloTrac also has the ability to track changes of CI compared with pulse contour analysis by PiCCO when they are induced by norepinephrine administration. 

Persistent or reoccurring shock, after initial resuscitation, is both an indication and recommendation for the monitoring of CI in critically ill patients [1]. Several different techniques are available to monitor CI. The choice of CI monitoring methods in individual critically ill patients depends on many factors such as patient condition, hemodynamic monitoring requirement, and monitoring the response to therapeutic interventions. 

Noncalibrated pulse contour analysis such as FloTrac/Vigileo, is one of the most popular used CI monitoring method for critically ill patients. Similar with our study, several studies showed that CI obtained by previous version of FloTrac had no acceptable agreement with the percentage error of 49–61%, when compared with transpulmonary thermodilution by PiCCO, [13–16]. Boettger et al. reported the bias of 0.72 L/min and limits of agreement of -2.16–3.6 L/min between FloTrac and thermodilution by PiCCO, and also demonstrated that FloTrac underestimation at high cardiac output but an overestimation at low cardiac output relative to transpulmonary thermodilution by PiCCO in septic patients [14]. Slagt et al. compared CI derived by the 3rd generation of FloTrac with transpulmonary thermodilution by VolumeView/EV1000 in sepsis patients [29]. They found moderate agreement between the two methods, with a percentage error of 48%, with poor to moderate CI tracking abilities. Moreover, the 3rd generation of FloTrac has a moderate ability to track changes in CI when compared with the thermodilution method [18,19].

The algorithm of FloTrac software was updated for improved performance of this device. The latest is the 4th version, and Kfast is the newly added component for faster response to the changes of the vascular tone. Two previous studies found that the performance along with trending ability of the 4th generation of FloTrac was improved after phenylephrine bolus compared with 3rd software in cardiac surgical patients [21,22]. Nevertheless, in one study the percentage error was higher than the clinically acceptable range (55.4%), due to bias having been correlated with the SVRI [22]. Similar, with our study in septic shock patients, the bias between CIv and CItd was strongly associated with SVRI. Moreover, bias of the 4th generation of FloTrac was still influenced by SVRI much the same as in the previous version. Therefore, the FloTrac software has room for improvement in the performance of its software. 

Validation of CI monitoring devices should not only be based on its ability to measure absolute CI values, but also on their ability to track trending changes of CI. Serial changes in CI also provide valuable information to intensivists to allow them to cope with critical conditions and make adjustment to the therapeutic treatment. The ability of the 3rd of FloTrac software to rapidly detect CI changes has been reported in septic shock patients. Monnet et al. showed that the 3rd generation of FloTrac has moderate reliability for tracking changes in CI induced by fluid expansion and norepinephrine administration [19]. However, in our study, for the 4th generation of FloTrac, it demonstrated the reliability in following the changes in CI, and changes in CI induced by norepinephrine administration. This study presented the relatively high correlation coefficients between the changes in CIv and CIp, via high concordance rates. This demonstrated that the 4th generation of the FloTrac algorithm appears to have improvements in its tracking ability compare to the previous version. 

While the accuracy of the 4th generation of FloTrac has been evaluated in patients undergoing cardiac surgery, our study is the first validation of the updated FloTrac software in septic shock patients. However, some limitations of our study should be taken into consideration. First, we compared the 4th generation of FloTrac with transpulmonary thermodilution using this as a reference method. Several studies have shown that the transpulmonary thermodilution by PiCCO provides just as a reliable estimation of CI as the thermodilution method by PAC [5–7], even though it is not the gold standard or a perfect CI method. Second, we validated the trending ability of CI by FloTrac utilizing only pulse contour analysis obtained by PiCCO when increasing dosage of norepinephrine or fluid bolus, because the hemodynamic instability of our septic shock patients is limited to performed thermodilution method. Monnet et al. found that neither CIp nor its tracking ability for the changes of CI induced by volume expansion and norepinephrine administration, when compared with transpulmonary thermodilution by PiCCO for septic shock patients was reliable and accurate [13]. Finally, we used a percentage error of 45% as clinically acceptable, which differs from previous references of 30%. This percentage error was suggested by Critchley and Critchley in a metaanalysis paper published in 1999 based on precision for the reference method of ± 20% [33]. However, the precision of CI measurement can vary with the use of different techniques. Recent, metaanalysis of accuracy and precision of CI monitoring in critically ill patients presented that none of the 4 minimally invasive CI measurements met the criteria for acceptability of agreement, as suggested by Critchley and Critchley [30]. Therefore, they suggested that the percentage error in agreement of 45% represents a more realistic achievable precision in clinical practice [11,30]. However, the percentage errors of 4th generation of FloTrac in our study remain beyond the revised boundary for septic shock patients. 

In conclusion, the 4th generation of FloTrac has not clinically acceptable agreement to assess CI, when compared with the transpulmonary thermodilution method by PiCCO. However, the tracking ability of the last updated version of FloTrac is reasonable for clinically relevant changes in CI and demonstrated good reliable for assessing the CI changes induced by norepinephrine. 

## Acknowledgements 

This study was supported by a research grant from the Faculty of Medicine, Prince of Songkla University. The authors would like to thank Dr. Sarayut Geater from the Department of Internal Medicine, Faculty of Medicine, Prince of Songkla University, for his assistance with the statistical components of the article. The authors would also like to acknowledge Andrew Jonathan Tait from the International Affairs Department, Faculty of Medicine, Prince of Songkla University for his help in editing this manuscript.
